# Hybrid Molecules with Purine and Pyrimidine Derivatives for Antitumor Therapy: News, Perspectives, and Future Directions

**DOI:** 10.3390/molecules30132707

**Published:** 2025-06-23

**Authors:** Simona Iacob (Ciobotaru), Claudia-Simona Stefan, Aurel Nechita, Madalina-Nicoleta Matei, Elena-Lacramioara Lisa, Dana Tutunaru, Iuliu Fulga, Ana Fulga, Alina-Georgiana Cristea (Hohota), Oana-Maria Dragostin

**Affiliations:** Research Centre in the Medical-Pharmaceutical Field, Faculty of Medicine and Pharmacy, “Dunarea de Jos” University of Galati, 35 AL Cuza St., 800010 Galati, Romania; simona.ciobotaru98@yahoo.com (S.I.); nechitaaurel@yahoo.com (A.N.); madalina.matei@ugal.ro (M.-N.M.); elena.lisa@ugal.ro (E.-L.L.); dana.tutunaru@ugal.ro (D.T.); fulgaiuliu@yahoo.com (I.F.); ana.fulgaa@gmail.com (A.F.); alina.cristea@ugal.ro (A.-G.C.)

**Keywords:** purine, pyrimidines, antitumoral activity, hybrid molecule

## Abstract

Cancer is a leading cause of death globally, claiming millions of lives each year. Despite the availability of numerous anticancer drugs, the need for new treatment options remains essential. Many current therapies come with significant toxicity, lead to various side effects, or do not consistently deliver the expected therapeutic results. Purines and pyrimidines are fundamental building blocks of nucleic acids and play crucial roles in cellular metabolism and signaling. Recent advances in medicinal chemistry have led to the development and synthesis of various derivatives that exhibit selective cytotoxic effects against cancer cells while minimizing toxicity to healthy tissues. Purine and pyrimidine scaffolds, due to their well-established biological roles and structural versatility, have emerged as key pharmacophoric fragments in anticancer drug discovery. In recent years, the rational design of hybrid molecules incorporating these heterocycles has shown promise in overcoming drug resistance, improving target selectivity, and enhancing pharmacological profiles. Purine and pyrimidines scaffolds hold significant potential as foundations for novel antitumor drugs, with established representatives in cancer treatment, including 5-fluorouracil, cladribine, capecitabine, and several others. In addition, the article discusses the challenges and future developments of purine and pyrimidine derivatives and hybrid molecules as antitumor drugs and emphasizes the need for continued research to optimize their effectiveness and reduce side effects. Overall, the innovative use of these compounds represents a major advance in targeted cancer therapy and holds promise for improving the therapeutic efficacy of malignant diseases.

## 1. Introduction

Cancer remains the leading cause of mortality worldwide, affecting millions of individuals each year [1]. Despite decades of intensive research dedicated to the development of novel anticancer agents, it continues to pose a significant global health challenge [2]. Cancer presents in a diverse array of forms, including hematologic (blood) cancers [3], bone cancers [4], breast cancer [5], endocrine cancers [6], genitourinary cancers [7], gastrointestinal (digestive) cancers [8], respiratory cancers [9], and skin cancers [10]. Over the past two decades, cancer therapies have advanced substantially, with the development of more potent agents that offer improved safety profiles and enhanced molecular specificity. Despite these significant achievements, drug resistance remains a major obstacle in cancer treatment [11]. Virtually all targeted anticancer therapies eventually face resistance during clinical application [12]. Multiple mechanisms have been implicated in this phenomenon, including genetic and epigenetic alterations, gene amplification, the presence of cancer stem cells (CSCs), overexpression of efflux transporters, dysregulation of apoptotic pathways, and autophagy, among others. These adaptive responses not only limit the long-term efficacy of treatment but also contribute to cancer recurrence and progression [13]. In response, alternative therapeutic strategies have been increasingly explored in drug design and discovery [14]. Among these, heterocyclic compounds containing purine and pyrimidine scaffolds have garnered considerable attention due to their promising potential in targeting various cancer types [15,16]. Emerging evidence from studies published over the past decade highlights that the incorporation of purine and pyrimidine rings into synthetic compounds has led to the identification of highly potent anticancer molecules [17].

Purine and pyrimidine scaffolds play a crucial role in various biological processes. In nature, they predominantly occur as integral components of larger molecular structures [18], such as nucleosides and nucleotides, either in their free form or incorporated into polynucleotide chains, coenzymes, and other biomolecules [19]. Molecular hybridization represents an emerging strategy in drug design and development, involving the fusion of pharmacophoric elements from distinct bioactive compounds to generate novel hybrid molecules with enhanced affinity and therapeutic efficacy compared to the original drugs [20]. Moreover, this approach can lead to compounds with altered selectivity profiles, distinct and/or dual mechanisms of action, and a reduced incidence of undesirable side effects [21]. In medicinal chemistry, a pharmacophore is defined as the ensemble of steric and electronic features necessary to ensure optimal interactions with a specific biological target and to trigger or block its biological response. Both purine and pyrimidine rings have long been recognized as privileged scaffolds due to their ability to mimic natural nucleobases and engage in key molecular interactions.

The design of pharmacophoric hybrids, which combine two or more active moieties within a single molecular entity, has emerged as a powerful strategy in anticancer drug development. Such hybrids may provide improved pharmacodynamic selectivity [22], favorable pharmacokinetic properties (e.g., solubility, metabolic stability), and can sometimes overcome drug resistance by simultaneously modulating multiple molecular targets. In this context, purine and pyrimidine hybrids are particularly attractive due to their structural diversity, biological relevance, and versatility in conjugation chemistry [23].

This review focuses on the biological potential of hybrid molecules, with particular emphasis on those demonstrating anticancer activity within the realm of pharmaceutical compounds. Recent advancements in the development of these hybrid classes are concisely presented. The objective of this review is to provide a concise yet comprehensive overview of recent progress in the synthesis and pharmacological evaluation of hybrid molecules across various therapeutic areas, aiming to support the broader scientific community, including both seasoned researchers and newcomers, in the rational design and development of enhanced pharmacological agents.

In contrast to previously published reviews, our work provides a focused analysis of hybrid molecules incorporating purine and pyrimidine scaffolds. In particular, we classify the hybrids based on their structural connectivity (fused, linked, merged, or spaced) and discuss the recent advances in the last years. Furthermore, we highlight hybrid designs inspired by clinically approved agents, offering a translational perspective that bridges preclinical research and clinical application.

The purine and pyrimidine-based hybrid molecules included in this review were selected based on specific criteria to ensure relevance and scientific quality. The selection focused on compounds reported in peer-reviewed journals from 2018 to 2025, with an emphasis on molecules demonstrating anticancer activity either in vitro or in vivo. Additional inclusion factors involved the presence of clear hybrid structures, novelty of the pharmacophoric combination, and availability of data on synthesis or structure–activity relationships.

## 2. Clinically Approved Drugs Containing Pyrimidine and Purine Scaffolds

Many clinically approved anticancer drugs are built on purine or pyrimidine scaffolds, which act as pharmacologically validated cores capable of interfering with nucleic acid metabolism or enzyme function [24]. In this section, we present an overview of selected drugs that exemplify the therapeutic success of these heterocycles. This overview not only demonstrates their clinical relevance but also highlights their potential as starting points or core fragments in the design of novel hybrid molecules. In recent years, several hybrid systems have incorporated structural features of approved drugs such as 5-fluorouracil or 6-mercaptopurine to enhance potency, selectivity, or reduce resistance.

Nucleoside analogs of purine and pyrimidine bases represent a critical class of antimetabolites employed in cancer therapy [25]. Due to their structural similarity to natural nucleosides, these compounds directly interfere with nucleic acid synthesis, thereby disrupting essential processes such as DNA replication and RNA transcription [26]. Upon intracellular phosphorylation, nucleoside analogs can be incorporated into DNA or RNA, leading to structural and functional abnormalities that result in cell cycle arrest and ultimately trigger cancer cell death through apoptosis or necrosis [27]. This ability to mimic natural nucleosides while introducing fatal errors into the genetic material underpins their therapeutic efficacy in the treatment of various malignancies [28].

Pyrimidine analogues such as 5-fluorouracil [29] (5-FU), capecitabine [30], cytarabine [31], azacitidine [32], trifluridine [33], and gemcitabine [34] are cornerstone chemotherapeutic agents (Table 1). They function by mimicking natural nucleosides, thereby disrupting DNA and RNA synthesis, leading to cell cycle arrest and apoptosis. For instance, 5-FU is widely used against colorectal, breast, and gastric cancers [35]. Capecitabine, an oral prodrug of 5-FU, offers similar efficacy with improved patient compliance [36]. Cytarabine and gemcitabine are primarily employed in hematological malignancies and solid tumors, respectively [37]. The pyrimidine nucleus serves as a foundational structure in designing protein kinase inhibitors, targeting enzymes critical for cancer cell proliferation and survival [38]. Notably, several FDA-approved tyrosine kinase inhibitors (TKIs), such as imatinib, dasatinib, and nilotinib, are built upon pyrimidine cores [39]. These drugs selectively inhibit aberrant kinases like BCR-ABL, c-KIT, and PDGFR, which are involved in the pathogenesis of cancers such as chronic myeloid leukemia (CML) and gastrointestinal stromal tumors (GISTs). The pyrimidine scaffold plays a crucial role in facilitating binding to the ATP-binding site of these kinases, thereby blocking downstream signaling and inducing apoptosis in malignant cells [40]. Recent research emphasizes the development of dual-target kinase inhibitors based on pyrimidine scaffolds, aiming to enhance therapeutic efficacy and overcome drug resistance [41].

Purine analogs like mercaptopurine [59], clofarabine [60], nelarabine [61], fludarabine [62], and cladribine [63] are widely used antimetabolite drugs in chemotherapy (Table 2). They mimic natural purines and interfere with DNA synthesis, effectively halting cancer cell division and inducing cell death [64]. These drugs are particularly effective in leukemias and lymphomas [65]. Some purine derivatives act as protein kinase inhibitors, targeting enzymes involved in cancer cell growth and survival. For instance, purvalanol-A [66] and seliciclib [67] are known to inhibit cyclin-dependent kinases (CDKs), showing significant antitumor activity.

Recent reviews highlight that molecules incorporating purine and pyrimidine cores have strong anticancer potential, often with nanomolar IC_50_ values, indicating high potency. These compounds can selectively target cancer-specific receptors or enzymes.

## 3. Recent and Classic Advances in the Synthesis of Purine and Pyrimidine Hybrids

**Figure 1:**
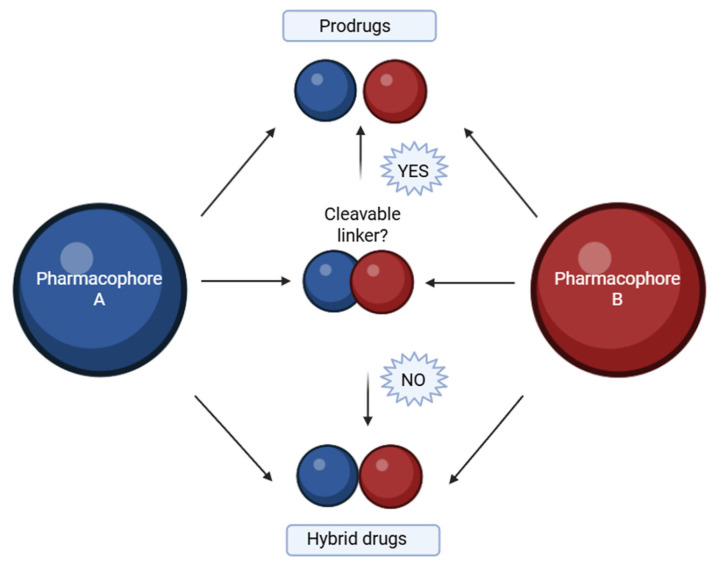
Base of hybrids molecules with cleavable linker or non-cleavable linker. Created in BioRender. Iacob (2025) https://BioRender.com/qv9ivu4.

A hybrid compound is a synthetically designed entity composed of two or more pharmacophores, each derived from known bioactive agents associated with a specific therapeutic effect [81]. Advances in medicinal chemistry have led to the development of such molecular hybrids, which are typically constructed by covalently linking distinct biologically active moieties [82]. By utilizing well-established template compounds, already characterized in terms of physicochemical properties, pharmacological profiles, toxicity, and mechanisms of action, it is possible to generate extensive chemical libraries comprising hundreds or even thousands of homologous molecular hybrids. These libraries offer a substantial body of accumulated knowledge, including insights into structural requirements, ligand–protein interaction mechanisms, binding site dynamics, and quantitative structure–activity relationships (QSAR). Such an approach significantly accelerates and enhances the efficiency of new drug development processes [83]. The goal is to preserve or enhance the pharmacological properties of each component within a single molecular framework. This linkage is generally achieved using a molecular linker that forms a covalent bond between the pharmacophores. Depending on the design, the linker can be either cleavable or non-cleavable (Figure 1). A cleavable linker is intended to undergo biotransformation at the target site, thereby releasing the active components in a prodrug-like manner. In contrast, a non-cleavable linker remains intact throughout the compound’s systemic circulation, representing a true hybrid drug strategy [84]. Hybrid molecules can be broadly categorized based on how the two pharmacophoric units are connected within the same molecular framework:

✓ Fused hybrids—the pharmacophores are directly fused into a single heterocyclic framework [85].
✓ Linked hybrids—the pharmacophores are connected via a stable linker (e.g., alkyl chain, amide bond) [86].
✓ Merged hybrids—overlapping atoms from both moieties are combined into one unified core [87].
✓ Spaced hybrids—pharmacophores are connected by a longer flexible spacer for dual-targeting [88].

Purine and pyrimidine scaffolds possess well-defined stereoelectronic properties that enable key interactions with biological targets (Table 3). The nitrogen atoms in their ring systems serve as hydrogen bond donors and acceptors, facilitating base-pairing, stacking, and metal coordination. Additionally, the planarity and electron-rich aromaticity of these heterocycles support π–π interactions and hydrophobic binding within nucleic acid- or protein-active sites. These features make purine and pyrimidine cores ideal as pharmacophores, particularly in anticancer therapy, where selective target engagement is critical [89]. Their modularity also allows facile hybridization with other pharmacophores, enabling dual or multitarget action.

### 3.1. Pyrimidine Hybrids and Various Derivatives with Antitumoral Activity

Pyrimidine, a nitrogen-containing heterocyclic compound, is well known for its broad spectrum of pharmacological activities, particularly its significant role in antitumor therapies [99].

**Figure 2:**
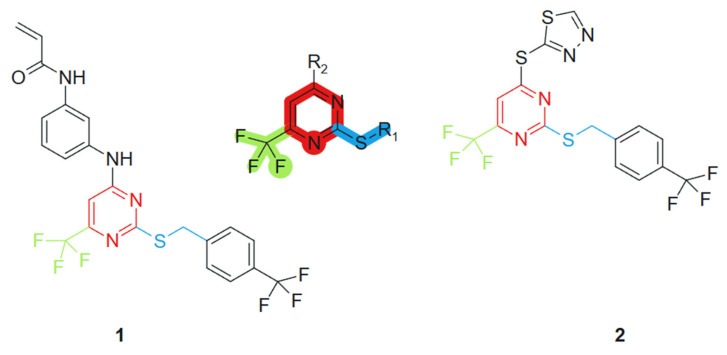
2-mercapto-6-trifluoromethylpyrimidine core scaffold, derivative molecules with antitumoral action.

Based on the studies of pyrimidine derivatives, Liu et al., 2021 [100] found that some compounds with better bioactivity have a sulfhydryl group at the 2-position of the pyrimidine ring or a trifluoromethyl group at the 6-position. Consequently, 2-mercapto-6-trifluoromethylpyrimidine was selected as the core scaffold for further investigation (Figure 2). Using the principle of molecular hybridization, acrylamide and trifluoromethyl groups were introduced into the pyrimidine framework to synthesize a series of novel pyrimidine compounds, whose biological activity was subsequently evaluated in vitro. The incorporation of the acrylamide moiety contributed to electrophilic reactivity and potential acceptor behavior, facilitating covalent interactions with biological targets [101]. The trifluoromethyl (CF_3_) group, a well-known bioisostere, increased the lipophilicity and metabolic stability of the hybrid, potentially improving membrane permeability [102]. These features, when combined with the purine scaffold, contributed to the observed synergistic activity. The acrylamide unit was introduced through standard amide coupling, while the CF_3_ group was incorporated via electrophilic trifluoromethylation under mild conditions. The following compounds were synthesized and assessed for their cytotoxic effects on four human tumor cell lines: PC-3 (prostate cancer), MGC-803 (gastric cancer), MCF-7 (breast cancer), and H1975 (non-small cell lung cancer). Among these, compounds **1** and **2** demonstrated significant antiproliferative activity against the H1975 cell line, with an IC_50_ value of 4.77 μM and 2.27 μM, respectively, outperforming the standard chemotherapeutic agent 5-fluorouracil (5-FU), which exhibited an IC_50_ of 9.37 μM under the same experimental conditions (Figure 2).

**Figure 3:**
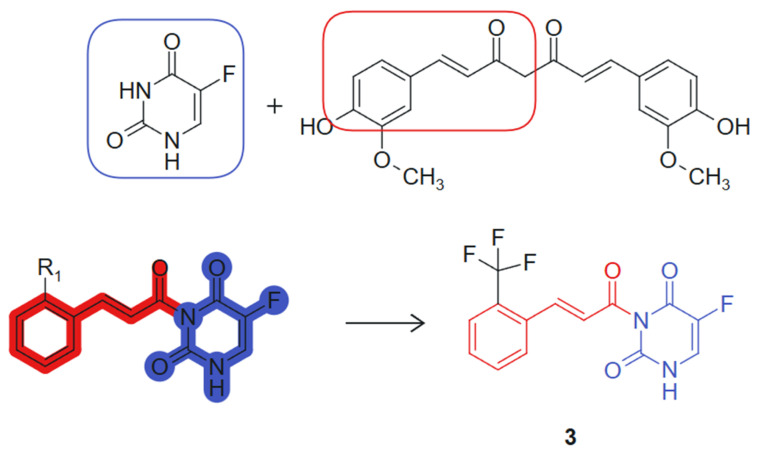
Curcumine-5-fluorouracil hybrid molecule with potential antitumoral action.

On the other hand, Wang et al., 2024 [103] designed and synthesized a series of novel curcumine-5-fluorouracil hybrids and determined their anti-cancer activity. This hybrid exemplifies a synergistic combination of two pharmacophores, a curcumin-derived chalcone unit (β-keto-enone, Michael acceptor) and 5-fluorouracil, an antimetabolite that inhibits thymidylate-synthase (TS) [104], each contributing distinct mechanisms of action: ROS generation and thioredoxin reductase inhibition from the curcumin moiety, and thymidylate synthase inhibition from the 5-FU fragment. The hybrid molecule benefits from stereoelectronic complementarity: The electron-rich conjugated system of curcumin supports π–π stacking and redox activity, while the planar pyrimidine ring of 5-FU enhances target binding through hydrogen bonding and base-mimicking interactions. The synthesized hybrid compounds underwent comprehensive in vitro cytotoxicity evaluations across a panel of cell lines, including A549 (human lung carcinoma), HepG2 (human hepatocellular carcinoma), HeLa (human cervical carcinoma), THLE-2 (normal human liver epithelial cells), and 4T1 (murine mammary carcinoma). Notably, several hybrids demonstrated significant antiproliferative activity against the cancer cell lines tested. Among these, compound **3**, linked hybrid with non-cleavable amide linker (Figure 3), exhibited the most potent antiproliferative activity against A549 lung cancer cells, with an IC_50_ value of 2.27 μM, surpassing the efficacy of 5-FU alone, which had an IC_50_ of 9.37 μM. Notably, compound **3** demonstrated high selectivity for cancer cells over normal THLE liver cells. In addition, mechanistic studies revealed that compound **3** targets thioredoxin reductase (TrxR), leading to increased levels of reactive oxygen species (ROS) within tumor cells and inducing apoptosis. This effect is attributed to the Michael acceptor moiety present in the curcumin structure. The two pharmacophores were joined via an amide linkage using a classical condensation strategy, preserving their bioactive cores while allowing structural flexibility. The presence of a Michael acceptor and the retention of the fluorinated pyrimidine ring were critical for enhancing biological activity [105]. Additionally, compound **3** inhibits thymidylate synthase (TS), causing cell cycle arrest at the G0/G1 phase, a property linked to the 5-FU component of the hybrid molecule. In vivo experiments further confirmed compound **3**’s efficacy, demonstrating significant reductions in tumor volume and weight in mice, with minimal toxic side effects. These findings suggest that the curcumin–5-FU hybrids holds promise as a lead compound for anticancer therapy, warranting further investigation.

**Figure 4:**
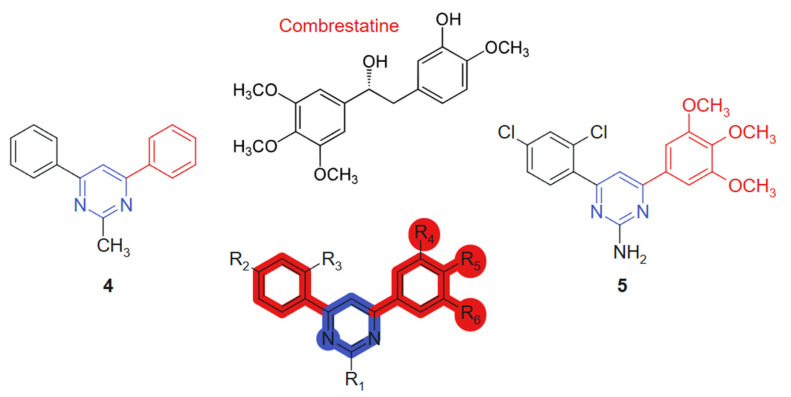
Pyrimidine-bridged combretastatin hybrid molecules with potential antitumor action.

Kumar et al., 2018 [106] evaluated, in their study, a novel series of pyrimidine-bridged combretastatin hybrid molecules, for anticancer activity against breast (MCF-7) and lung (A549) cancer cell lines using MTT assays. Among the tested compounds, two linked hybrids (direct aryl-hetero ring bonds, non-cleavable) (Figure 4) exhibited notable antiproliferative effects. The compound **4** showed IC_50_ values of 4.67 µM (MCF-7) and 3.38 µM (A549), while the compound **5** demonstrated IC_50_ values of 4.63 µM and 3.71 µM against MCF-7 and A549 cell lines, respectively. The hybrid design combines the well-established tubulin-binding pharmacophore of combretastatin [107] with a pyrimidine bridge, aiming to enhance target interaction and improve metabolic stability. The observed cytotoxicity suggests synergistic effects derived from dual pharmacological action. These results indicate strong cytotoxic potential, particularly against lung cancer cells. Their efficacy is likely attributed to the inhibition of antioxidant enzymes and the subsequent elevation of intracellular reactive oxygen species (ROS), which activate the intrinsic apoptotic pathway. The evaluated compounds demonstrated no detectable cytotoxicity toward normal human primary cells. The incorporation of a planar pyrimidine linker contributes to the overall rigidity and alignment of pharmacophoric groups, enhancing π–π interactions. Methoxy-substituted aromatic rings from the combretastatin moiety provide additional electronic density for optimal receptor engagement [108]. Significantly, compound **4** was identified as a competitive inhibitor of colchicine, exhibiting comparable potency in suppressing tubulin polymerization in vitro. Binding assays further revealed that its inhibitory activity closely mirrored that of colchicine. Complementary molecular modeling studies corroborated these findings, confirming that the synthesized compounds exhibit optimal steric and electronic complementarity within the colchicine-binding pocket of tubulin.

**Figure 5:**
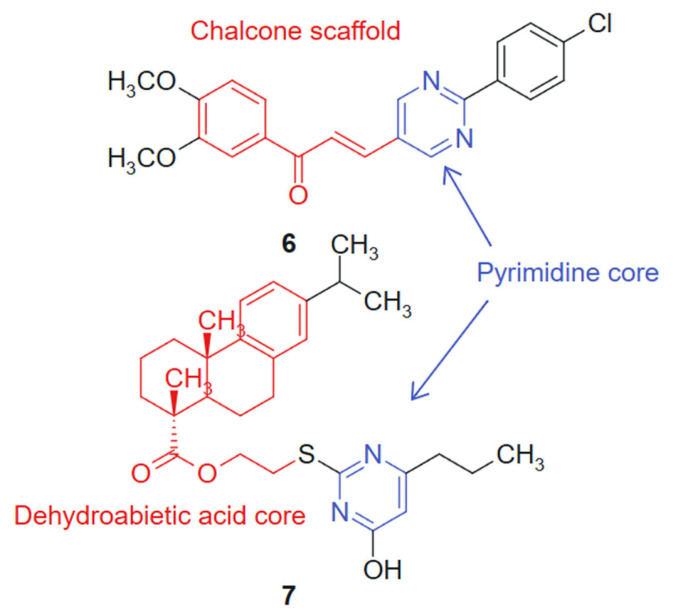
Pyrimidine-chalcone and pyrimidine-dehydroabietic acid targeted agents.

In another study Reymova et al., 2025 [109] synthesized a novel series of pyrimidine-tethered chalcone hybrids and evaluated their anticancer potential. Among the tested compounds, compound **6** linked hybrid-non-cleavable vinyl/alkenyl linker. (Figure 5) demonstrated notable cytotoxicity against MCF-7 breast cancer cells (IC_50_ = 6.70 ± 1.02 µM) and A549 non-small cell lung cancer (NSCLC) cells (IC_50_ = 20.49 ± 2.7 µM), performing comparably or better than the standard EGFR inhibitor lapatinib. Compound 6 also showed selectivity toward leukemic Jurkat T cells over healthy peripheral blood mononuclear cells (PBMCs), suggesting a therapeutic window. In silico pharmacokinetic profiling indicated that compound 6 exhibits drug-like properties, reinforcing its potential as a lead compound. The hybridization of the pyrimidine ring, a recognized antimetabolite scaffold [110], with the α,β-unsaturated ketone of the chalcone moiety was designed to combine antiproliferative and apoptosis-inducing properties within a single molecular framework. The activity profile of compound **6** reflects a synergistic effect, where both fragments contribute complementary mechanisms of action. The chalcone moiety introduces a planar, conjugated system that can participate in Michael-type interactions with cellular nucleophiles, while the pyrimidine ring offers hydrogen bond acceptors and donors for binding to kinase or nucleotide-binding pockets. This duality supports the observed selectivity and potency [111].

In addition, a series of novel dehydroabietic acid derivatives incorporating pyrimidine moieties was designed and synthesized by Huang et al., 2020 [112] to develop more effective and less toxic antitumor agents, based on the principles of molecular combination and hybridization. The design strategy relies on a synergistic hybridization between dehydroabietic acid, a diterpenoid with known anti-inflammatory and cytotoxic effects [113], and the pyrimidine scaffold, a well-established pharmacophore in anticancer therapy. This molecular fusion aimed to combine the lipophilic, membrane-interacting properties of the tricyclic acid with the hydrogen bonding and enzyme-targeting capacity of the pyrimidine ring. The cytotoxicity of these compounds was evaluated against human liver cancer (HepG2) cells, human breast cancer (MCF-7) cells, human colon cancer (HCT-116) cells, human lung cancer (A549) cells, and human normal liver cells (LO2) using the MTT assay in vitro. Cytotoxicity screening revealed that most of the compounds exhibited moderate to high levels of cytotoxicity against the four cancer cell lines, with some displaying more potent inhibitory activities compared to the standard chemotherapeutic agent 5-fluorouracil (5-FU). Notably, compound **7**, linked hybrid- non-cleavable thioether/alkyl linker (Figure 5), demonstrated promising cytotoxicity, with IC50 values ranging from 7.00 to 11.93 μM against all the tested cancer cell lines, while showing weak cytotoxicity towards normal cells. Furthermore, cell cycle analysis indicated that compound **7** primarily induced a cell cycle arrest at the S-phase in MCF-7 cells and triggered apoptosis. The synthetic approach employed standard esterification and amidation reactions to conjugate pyrimidine moieties to the dehydroabietic acid core, preserving both pharmacophores. This modular strategy allowed the efficient assembly and diversification of the hybrid series for SAR optimization [114].

**Figure 6:**
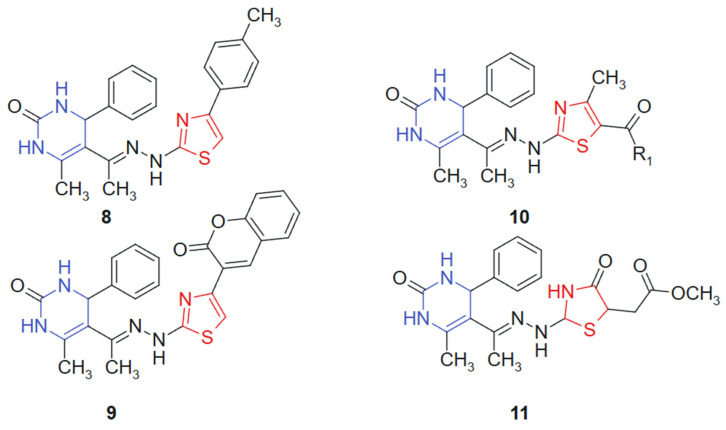
1,3-thiazolyl–pyrimidine derivatives-potential anticancer agents.

A series of novel 1,3-thiazolyl–pyrimidine derivatives was synthesized and structurally characterized by Abolibda et al., 2025 [115] using spectroscopic techniques to evaluate their potential as anticancer agents. The cytotoxic activity of these compounds was assessed against HepG2 human liver cancer cells using the MTT assay. Among the tested molecules, linked hybrids non-cleavable hydrazide linker compounds **8**, **9**, **10a** (R_1_ = -CH_3_), **10b** (R_1_ = -OCH_2_CH_3_), and **11** (Figure 6) demonstrated notable antiproliferative effects, with IC_50_ values of 5.02 ± 1.83, 4.04 ± 1.37, 3.81 ± 1.96, 2.39 ± 0.75, and 3.27 ± 1.13 μM, respectively. Remarkably, all five compounds exhibited greater cytotoxic potency compared to the standard reference drug doxorubicin (IC_50_ = 6.18 ± 0.29 μM). The hybridization of the pyrimidine core with a 1,3-thiazole moiety was designed to unite two pharmacophoric systems with documented anticancer potential. The pyrimidine ring mimics nucleobase structures and engages with nucleotide-binding sites, while the thiazole ring contributes to target selectivity and redox reactivity, creating a synergistic molecular platform for cytotoxic activity [116]. The resulting hybrid structure displays a planar conformation conducive to π–π stacking with DNA or protein residues, along with heteroatom-rich regions (N and S) for hydrogen bonding and dipolar interactions. Substituent variation at the R_1_ position influenced electronic distribution, with electron-donating groups (-CH_3_ and -OCH_2_CH_3_) enhancing bioactivity, likely due to improved lipophilicity and membrane permeability [117].

### 3.2. Purine Hybrids and Various Derivatives with Antitumoral Activity

Purine, a nitrogen-containing fused heterocyclic compound, is recognized for its pivotal role in numerous biological processes and its wide array of pharmacological activities. Owing to its structural versatility and ability to interact with various biological targets, purine and its derivatives have shown remarkable potential in the development of therapeutic agents, particularly in anticancer, antiviral, and anti-inflammatory treatments [118].

**Figure 7:**
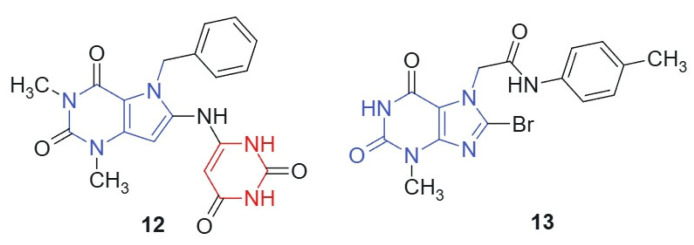
Purine scaffold for potential anticancer hybrid agents.

Mohamed et al., 2021 [119] synthesized a novel series of pyrimidine-containing purine analogs as potential anticancer agents. All compounds were evaluated for their cytotoxic activity against the NCI-60 human tumor cell line panel using the MTT assay. The results revealed that compound **12** and compound **13**, linked hybrids-non-cleavable amide linker (Figure 7), exhibited promising broad-spectrum anticancer activity, with a mean growth inhibition (GI) percentage of 41%. Notably, both compounds demonstrated potent activity (100% GI) against several cancer cell lines, including renal (A498), breast (MDA-MB-435, Hs 578T), colon (COLO 205, HT29), brain (SNB-75), and ovarian (OVCAR-3). The enhanced cytotoxic activity of compounds **12** and **13** may arise from the synergistic integration of two pharmacophores: the purine core, which is known to interact with kinases, DNA, and other nucleotide-binding proteins [120], and the barbituric acid moiety, a well-established hydrogen bond donor/acceptor system found in multiple bioactive scaffolds [24]. The conjugated and semi-planar geometry of the hybrid facilitates π–π stacking and potential intercalative interactions with nucleic acids or protein aromatic residues. In addition, the electron distribution across the purine–ureide framework may enhance binding affinity through favorable stereoelectronic complementarity with target sites. The strategic substitution at C-8 of the purine ring via nucleophilic displacement ensures the structural integrity and bioavailability of the hybrid scaffold. These features likely contribute to the observed broad-spectrum anticancer activity, particularly against renal, brain, and colon cancer cell lines.

**Figure 8:**
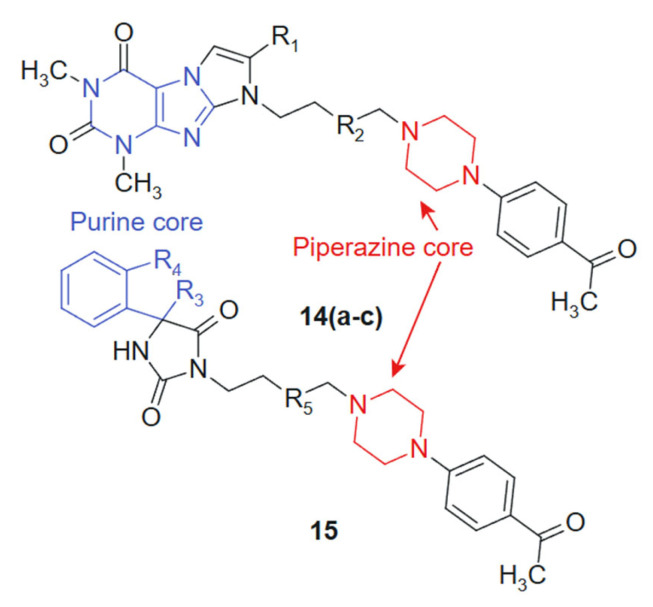
Purine-Piperazine Hybrids as Promising Anticancer Agents.

In their quest to develop novel anticancer therapeutics, Zagórska et al., 2021 [121] synthesized and biologically evaluated two novel series of hydantoin ((**14a**–**c**) **14a**: R_1_ = -H; R_2_ = -CH_2_; **14b**: R_1_ = -CH_3_; R_2_ = -CH_2_; **14c**: R_1_ = -H; R_2_ = -CH_2_-CH_2_) and purine ((**15**) R_3_ = -CH_2_; R_4_ = -CH_2_-CH_2_; R_5_ = -CH_2_-CH_2_) derivatives featuring a 4-acetylphenylpiperazinylalkyl pharmacophore (Figure 8). The purine core, known for its base-mimicking and enzyme-targeting capacity, was hybridized with a 4-acetylphenylpiperazinylalkyl moiety to enhance selectivity and improve membrane permeability. The combined structural features are designed to interact simultaneously with nucleoside metabolism pathways and secondary receptors involved in tumor proliferation, resulting in a synergistic anticancer profile. The purine ring contributes planarity and electron-rich sites capable of hydrogen bonding and π-stacking with nucleotide-binding enzymes, while the flexible alkyl-piperazine chain allows adaptation within hydrophobic or amphipathic receptor environments. This complementarity may underlie the improved selectivity indices observed in tumor versus normal cells [122]. The MTT-based screening against prostate (PC3) and colon (SW480, SW620) cancer cell lines identified compound **15** as the most potent candidate, exhibiting IC50 values of 16.8 μM (SW480), 12.9 μM (SW620), and 20.58 μM (PC3), while demonstrating superior tumor selectivity with selectivity indices of 8.88, 11.57, and 7.25 against these respective cell lines compared to normal HMEC1 cells. The hydantoin analogs **14a**–**c** showed preferential activity against PC3 cells (IC50 = 60.28–101.26 μM). Additional pharmacological profiling revealed that derivatives displayed negligible hemolytic effects on human erythrocytes at 10^−6^ M and inhibited thymidine phosphorylase by 21.21% at 100 μM, suggesting a potentially favorable safety profile combined with moderate enzymatic inhibition capacity that warrants further mechanistic investigation [123].

**Figure 9:**
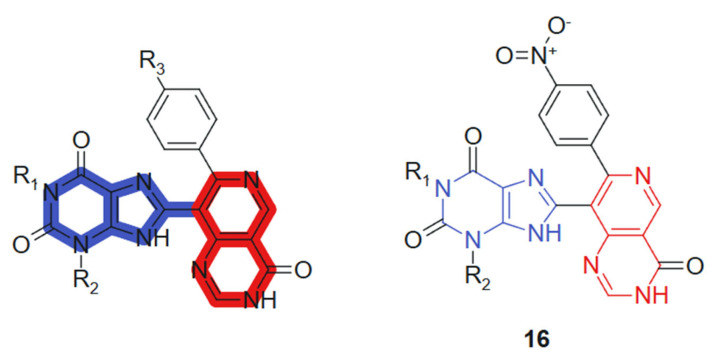
Purine-pyrimidine hybrid molecule for antitumoral action.

In another recent study, Verma et al., 2022 [124] reported the synthesis of 1,3-dimethyl-1H-purine-2,6(3H,9H)-dione derivatives conjugated with pyridopyrimidine, pyrazolopyridine, and pyranonaphthyridine ring systems. All synthesized compounds were evaluated for their anticancer activities. In vitro cytotoxicity was assessed using the MTT assay promising anticancer activity against a panel of human cancer cell lines, including MCF-7 (breast), A549 (lung), HeLa (cervical), and PANC-1 (pancreatic). Among the tested compounds, compound **16** (Figure 9) exhibited notably higher cytotoxic activity against MCF-7 (IC_50_ = 0.8 ± 0.61 µM), A-549 (IC_50_ = 1.0 ± 0.3 µM), and HeLa (IC_50_ = 1.2 ± 0.7 µM) cell lines. Additionally, it demonstrated comparable potency against Panc-1 cells (IC_50_ = 0.90 ± 0.71 µM) to that of the standard anticancer drug doxorubicin, whose IC_50_ values were 0.92 ± 0.50, 1.02 ± 0.80, 1.02 ± 0.72, and 1.41 ± 0.58 µM, respectively. These hybrids incorporate a purine core conjugated with various heterocyclic systems such as pyridopyrimidine, pyrazolopyridine, and pyranonaphthyridine. The synergy arises from combining the well-established bioactivity of methylxanthines, particularly their DNA-intercalative and phosphodiesterase-inhibitory potential, with the kinase-targeting and DNA-binding capacity of the fused heterocycles. The purine core contributes planarity, hydrogen bonding capability via carbonyl and NH groups, and lipophilicity, whereas the extended heterocyclic substituents provide π-conjugated systems that favor strong stacking and van der Waals interactions [125]. Substituents modulate the electronic density of the conjugate system, enhancing selectivity and potency.

**Figure 10:**
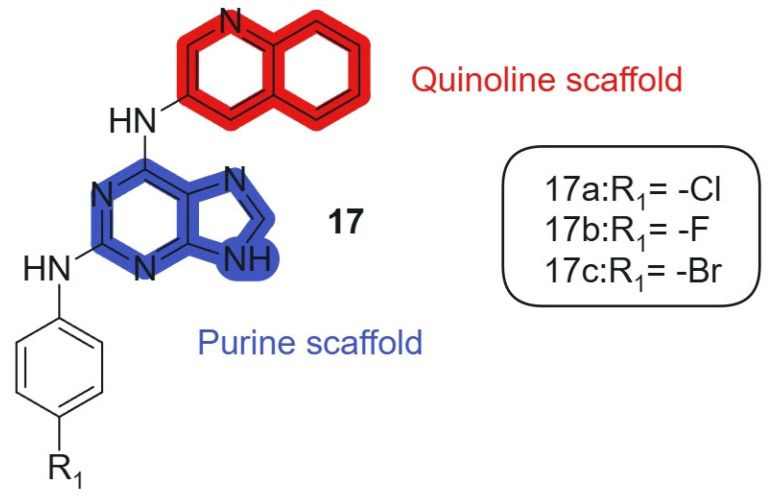
Purine-quinoline hybrid molecules for anticancer therapy.

Also, Kapadiya et al., 2018 [126] synthesized 15 purine-hybridized quinoline derivatives in two steps and evaluated their cytotoxicity against NCI-60 cell lines. Regioselective structural analysis using HMBC NMR spectra indicated that the C-6 position of purine was preferred over C-2 due to its electron-deficient center. The synthesized compounds were tested in a single-dose assay, revealing significant GI50 values. The selected compounds, **17a**, **17b**, and **17c** (Figure 10), linked hybrids-non-cleavable secondary-amine linker showed potent cytotoxicity, with compound **17a** being the most effective (GI50 = 7.57 μM) against the HOP-92 cell line, compared to cisplatin (GI50 = 1.4 μM). Furthermore, all compounds exhibited moderate to strong activity against prostate cancer (PC-3) cell lines. Overall, compound **17a** emerged as the most selective, showing considerable cytotoxic activity against HOP-92 and promising results against EKVX and PC-3 cell lines. The hybridization of the purine core with a quinoline fragment was intended to combine nucleobase mimicry and DNA intercalation potential within a single scaffold. The quinoline moiety enhances lipophilicity and stacking interactions with nucleic acids, while the purine offers key H-bonding functionalities, enabling dual pharmacophoric targeting. The electron-deficient character of the C-6 position in the purine ring allows optimal alignment of the quinoline system, maintaining the conjugation and planar electronic architecture necessary for π–π interactions within DNA and enzyme binding pockets.

**Figure 11:**
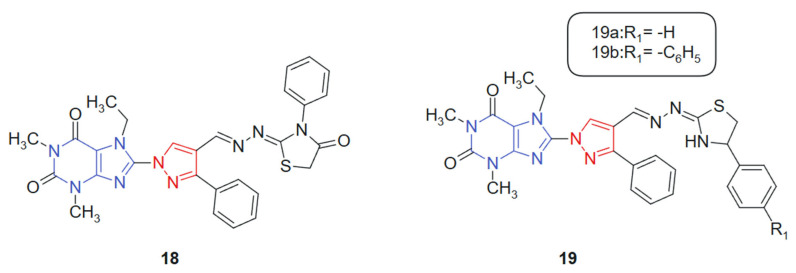
Purine–pyrazole hybrids as potential anticancer agents.

Also, with the idea of obtaining new hybrids, Afifi et al. 2019 [127] reported the synthesis of four novel series of purine–pyrazole hybrids incorporating thiazole, thiazolidinone, and rhodanine scaffolds and evaluated their anti-inflammatory, antioxidant, and anticancer potential. All synthesized compounds were screened against A549, Caco-2, PC-3, MCF-7, and HepG-2 cancer cell lines using the MTT assay, with 5-fluorouracil (5-FU) as the reference drug. Compounds **18**, **19a**, and **19b** (Figure 11) exhibited notable cytotoxicity, with IC_50_ values ranging from 18.85 to 238.11 μM, outperforming 5-FU (IC_50_ = 82.26–112.24 μM) across the tested cell lines. Notably, compound **18** emerged as the most potent, demonstrating IC_50_ values between 18.50 and 23.43 μM against all cell lines. Structure–activity relationship (SAR) analysis revealed that compounds featuring the rhodanine moiety were largely inactive, while those containing thiazoline groups showed superior cytotoxic profiles. This hybrid system was designed to integrate multiple bioactive fragments, purine, pyrazole, and thiazole-derived moieties, into a single framework, aiming to enhance biological efficacy through multi-target synergy. The purine ring contributes nucleobase mimicry, while the pyrazole and thiazole scaffolds add antioxidant and kinase-inhibitory properties. The purine moiety offers planar aromaticity and key nitrogen sites for hydrogen bonding, whereas the thiazole and thiazolidinone rings introduce additional π-conjugation and heteroatoms that promote polar interactions within active sites.

**Figure 12:**
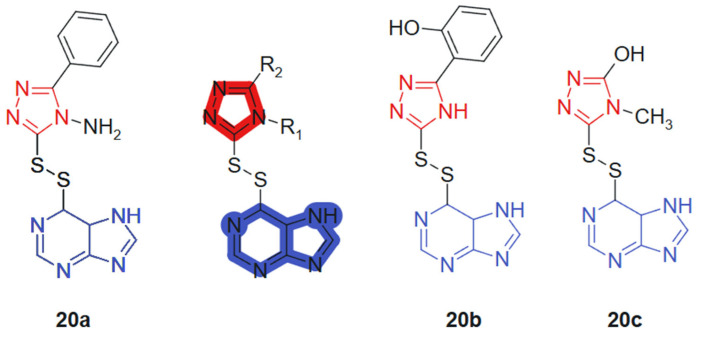
Purine-hybridized triazole derivatives.

Al-Duhaidahwi et al., 2018 [128] reported the design, synthesis, and biological evaluation of a novel series of substituted triazole-appended purine derivatives connected via a disulfide linker, developed as potential anticancer agents. The anticancer activity of compounds **20a**–**20c**, linked hybrids-cleavable disulfide linker, was assessed through MTT assay against three leukemia cell lines: rat lymphocytic leukemia (L1210), human promyelocytic leukemia (HL60), and human lymphoblastic leukemia (CCL-119). The results revealed that compounds **20a**, **20b**, and **20c** (Figure 12) exhibited potent antiproliferative activity, with IC_50_ values of 11.9, 9.8, and 7.4 μM (L1210); 6.2, 12.2, and 10.4 μM (HL60); and 8.3, 8.6, and 5.3 μM (CCL-119), respectively. These values demonstrated superior or comparable efficacy to the reference drug 6-mercaptopurine, which showed IC_50_ values of 9.7 μM, 11.3 μM, and 12.4 μM against L1210, HL60, and CCL-119, respectively. The hybrid design leverages the well-known pharmacological potential of purine scaffolds and the bioisosteric properties of 1,2,3-triazoles, while introducing a disulfide bridge to enable redox-responsive behavior in cancer cells. This tri-component hybrid structure enhances activity via synergistic interactions: purine for base mimicry, triazole for metabolic stability and π-stacking, and the disulfide linker for controlled intracellular release in the reductive tumor environment.

**Figure 13:**
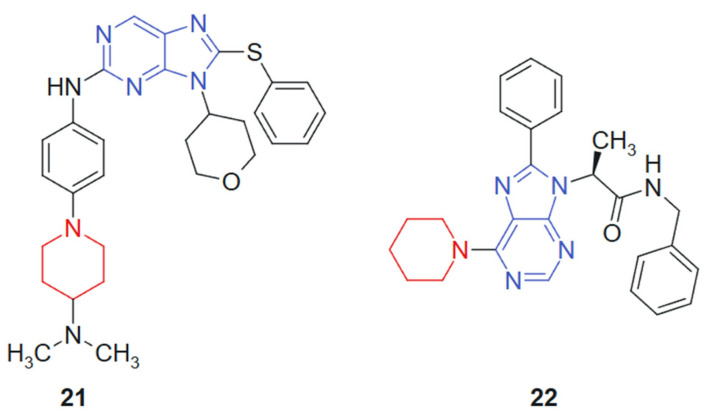
Purine–piperidine hybrids as potential anticancer agents.

Hei et al., 2019 [129] reported the synthesis and biological evaluation of a novel series of EGFR inhibitors incorporating a 2,9-disubstituted 8-phenylthio phenylsulfinyl-9H-purine scaffold. A total of 31 compounds were synthesized and screened for anticancer activity. Among them, compound **21** (Figure 13) demonstrated potent activity with an IC_50_ of 29.4 nM against the HCC827 lung cancer cell line and 1.9 nM against the mutant EGFR^L858R. Western blot analysis further confirmed that compound **21** effectively inhibited EGFR phosphorylation. In an in vivo xenograft model using HCC827 tumor-bearing nude mice, compound **21** significantly suppressed tumor growth when administered orally at a dose of 5.0 mg/kg. The hybrid scaffold integrates a purine nucleus with phenylthio or phenylsulfinyl and is specifically designed to optimize binding within the EGFR ATP-binding pocket. The combination enhances molecular recognition through a synergistic effect between the hydrogen-bonding capacity of the purine ring and the hydrophobic/polar interactions conferred by the sulfur-containing side chains.

Vanda et al. 2018 [130] reported the solid-phase synthesis of novel N-9-substituted amino acids and C-6-substituted piperidine analogs. Due to poor solubility, only five of the synthesized compounds were evaluated for anticancer activity against MCF-7 and K562 cancer cell lines using the Calcein AM assay. Among them, compound **22** (Figure 13) demonstrated the most promising cytotoxic effects, with IC_50_ values of 53 μM against K562 and 88 μM against MCF-7. Mechanistic investigations using flow cytometry revealed that treatment with compound **22** led to a significant, dose-dependent inhibition of cell proliferation, primarily through G1-phase cell cycle arrest and increased caspase activity, suggesting apoptosis induction. The hybrid system is based on the combination of a purine scaffold substituted at N-9 with amino acid derivatives and at C-6 with a piperidine ring. This design merges nucleobase mimicry with flexible side chains capable of modulating hydrophilicity and receptor binding, suggesting a synergistic contribution from both regions in enhancing cytotoxic potential. The purine ring provides an electron-rich, planar structure favoring hydrogen bonding and π–π stacking interactions, while the piperidine moiety introduces conformational flexibility and potential ionic interactions. The presence of amino acid substituents contributes to aqueous solubility and, possibly, to transporter-mediated uptake, although limited solubility remained an issue.

In recent years, modern synthetic approaches have been increasingly employed to develop purine and pyrimidine-based hybrid molecules. These include microwave-assisted synthesis [131], one-pot multicomponent reactions [132], green chemistry protocols [133], and the use of sustainable metal catalysts [134]. Other studies have reported the application of Biginelli-type multicomponent reactions [135] for the synthesis of pyrimidine hybrids under solvent-free or environmentally benign conditions, showing improved efficiency and atom economy. Additionally, microwave-promoted reactions have demonstrated significant time reduction and yield enhancement in the construction of both purine- and pyrimidine-based heterocycles. These modern methodologies offer advantages over traditional routes, including shorter reaction times, higher selectivity, and improved environmental compatibility.

For instance, an efficient one-pot synthesis of pyrimido[4,5-d]pyrimidine derivatives has been developed by Sahrapeyma et al., 2024 using a novel DABCO-based ionic liquid catalyst, [C_4_(DABCO-SO_3_H)_2_]·4ClO_4_ [136]. This method is notable for its short reaction times, its high product yields, and the straightforward isolation of products from the reaction medium. Additionally, the catalyst exhibits excellent reusability, contributing to the overall sustainability of the process [136].

Also, a novel series of fluorinated fused-pyrimidine derivatives, including pyrazolopyrimidines, triazolopyrimidines, and pyrimidobenzimidazoles, was designed and synthesized by Alnaja et al., 2021 using both conventional thermal methods and microwave irradiation techniques [137]. The proposed mechanistic pathways and the structures of all synthesized compounds were thoroughly investigated and confirmed through comprehensive spectroscopic analyses. The in vitro cytotoxic activity of the newly prepared compounds was evaluated against three human cancer cell lines: hepatocellular carcinoma (HepG-2), breast adenocarcinoma (MCF-7), and colorectal carcinoma (HCT-116), in order to assess their antitumor potential [137].

On the other hand, Katiya et al., 2025 reported the synthesis of pyrimidine and fused pyrimidine derivatives using a multicomponent telescopic reaction strategy that offers both high efficiency and environmental sustainability [138]. In their protocol, a one-pot reaction was carried out among barbiturates, aromatic aldehydes, hydrazine hydrate, phenyl isothiocyanate, and isoniazid under mild stirring at room temperature. This telescopic approach significantly reduced reaction time, avoided waste generation, and minimized operational complexity, in full accordance with green chemistry principles. Furthermore, the reactions were conducted in a green solvent system, and the resulting compounds were optimized using density functional theory (DFT) calculations at the B3LYP/6-31G(d,p) level to assess their physicochemical properties. These results highlight the potential of telescopic multicomponent strategies in the development of sustainable heterocyclic frameworks [138].

## 4. Conclusions and Future Perspectives

The ongoing global burden of cancer underscores the urgent need for more effective and safer therapeutic options. While numerous anticancer agents are currently available, their limitations, such as significant toxicity, adverse side effects, and inconsistent clinical outcomes, highlight the necessity for continued innovation in drug development. Purine and pyrimidine derivatives, as core scaffolds of nucleic acids, have demonstrated considerable promise in this regard. Their fundamental biological roles provide a rational basis for the selective targeting of rapidly proliferating cancer cells, while sparing normal tissues. This review has emphasized the diverse mechanisms of action exhibited by purine and pyrimidine-based compounds, ranging from DNA synthesis inhibition to interference with key metabolic and signaling pathways. Importantly, several clinically approved drugs based on these scaffolds, such as 5-fluorouracil and cladribine, have validated the therapeutic potential of these molecules.

In addition, we outlined recent advances in the design, synthesis, and biological profiling of hybrid molecules bearing purine and pyrimidine scaffolds for anticancer therapy. Through the analysis of representative compounds, we highlighted that structural hybridization enhances not only cytotoxic potency but also selectivity and mechanistic versatility. Several compounds, such as compound **21** (a purine-based EGFR inhibitor) and compound **16** (a purine–pyridopyrimidine hybrid), exemplify how rational scaffold integration can yield highly potent anticancer agents. Similarly, compound **3**, a curcumin–5-FU hybrid, showed remarkable activity and selectivity toward lung carcinoma cells.

By classifying the hybrid structures into fused, linked, and merged types, and analyzing their pharmacophoric synergy, stereoelectronic properties, and synthetic strategies, we provide a framework for understanding how molecular design impacts the bioactivity. The purine and pyrimidine cores confer planarity, hydrogen bonding potential, and modifiable positions that allow fine-tuning of pharmacokinetics and target interactions. Taken together, these findings confirm that hybrid molecules incorporating nucleobase-derived scaffolds represent a promising direction in anticancer drug discovery. Further exploration of their pharmacokinetic behavior, target selectivity, and scaffold-linker combinations is warranted to advance these leads into clinical candidates.

This review has systematically analyzed recent developments in their synthesis and biological evaluation, particularly focusing on compounds reported in the last years. These hybrids, by integrating multiple pharmacophoric motifs into a single framework, offer the potential to achieve improved selectivity, enhanced potency, and, possibly, reduced side effects compared to conventional monotherapies. Despite these advances, several challenges remain valid, such as drug resistance, off-target effects, and limited bioavailability, which continue to impede the full clinical potential of purine and pyrimidine analogs. Moreover, the complex interplay between tumor heterogeneity and the tumor microenvironment necessitates a more nuanced approach to drug design.

To address these issues, future research should focus on the following:

✓ Structural optimization of purine and pyrimidine derivatives and their hybrids, to improve specificity and reduce toxicity;
✓ Combination therapies that leverage synergistic mechanisms, potentially overcoming resistance and enhancing efficacy;
✓ Targeted delivery systems to increase drug accumulation within tumors while minimizing systemic exposure;
✓ Advanced screening techniques and computational modeling to predict drug behavior and guide rational design;
✓ Investigation of molecular mechanisms underlying drug resistance to inform the development of next-generation analogs.

The innovative hybrid molecules and derivatives discussed herein not only reinforce the relevance of nucleic acid-based frameworks but also pave the way for improved outcomes in the fight against malignant diseases.

In conclusion, purine and pyrimidine derivatives, along with their hybrid molecules, represent a promising frontier in the development of novel antitumor agents. Their structural versatility and ability to interfere with vital cellular processes such as DNA replication, transcription, and enzyme activity make them valuable scaffolds for anticancer drug design. As research continues to uncover the molecular mechanisms underlying tumor biology, the rational design of new purine- and pyrimidine-based hybrids holds significant potential to yield more effective and personalized cancer therapies.

## Figures and Tables

**Table 1:** Clinically approved drugs containing 5 FU and pyrimidine scaffold.

Drug	Structure	Mechanism of Action	Clinical Indication	Refs.
5-flourouracil	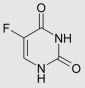	5-FU is metabolized intracellularly to 5-fluoro-2′-deoxyuridine monophosphate (FdUMP), which inhibits thymidylate synthase (TS), leading to disruption of DNA synthesis.	Colorectal, breast, gastric, head and neck cancers	[42,43,44,45,46]
Capecitabine	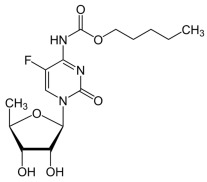	Capecitabine is enzymatically converted to 5-FU in tumor tissues. The resulting 5-FU then inhibits thymidylate synthase and incorporates into RNA and DNA, disrupting their synthesis and function.	Metastatic colorectal cancer, breast cancer	[47,48]
Gemcitabine	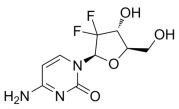	Gemcitabine is phosphorylated to its active diphosphate and triphosphate forms. The diphosphate inhibits ribonucleotide reductase, reducing deoxynucleotide pools, while the triphosphate incorporates into DNA, causing chain termination and apoptosis.	Pancreatic, non-small cell lung, bladder and breast cancers	[49,50,51,52]
Cytarabine	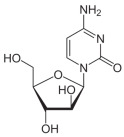	Cytarabine is converted to ara-CTP, which inhibits DNA polymerase and incorporates into DNA, leading to chain termination and inhibition of DNA synthesis.	Acute myeloid leukemia (AML)	[53,54]
Azacitidine	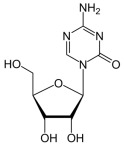	Azacitidine incorporates into RNA and DNA, inhibiting DNA methyltransferase, leading to hypomethylation of DNA and reactivation of tumor suppressor genes.	Myelodysplastic syndromes (MDS), acute myeloid leukemia (AML)	[55,56]
Trifluridine	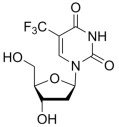	Trifluridine is incorporated into DNA, leading to DNA dysfunction and strand breaks. While it also inhibits thymidylate synthase, its primary cytotoxic effect is through DNA incorporation.	Metastatic colorectal cancer refractory to standard therapies	[57,58]

**Table 2:** Clinically approved drugs containing mercaptopurine and purine scaffold compounds.

Drug	Structure	Mechanism of Action	Clinical Indication	Refs.
Mercaptopurine	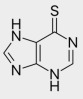	Mercaptopurine is converted intracellularly to thioinosinic acid, which inhibits several enzymes involved in purine metabolism. This inhibition disrupts DNA and RNA synthesis, leading to cytotoxicity.	Acute lymphoblastic leukemiaAcute myeloid leukemia	[68,69]
Cladribine	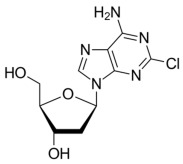	Cladribine is phosphorylated intracellularly to its active form, which inhibits DNA synthesis and repair by incorporating into DNA and inhibiting enzymes involved in DNA metabolism.	Hairy cell leukemiaNon-Hodgkin lymphoma	[70,71,72]
Clofarabine	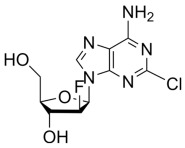	Clofarabine inhibits DNA polymerases and ribonucleotide reductase. It induces apoptosis in both cycling and non-cycling cells by disrupting DNA synthesis and repair mechanisms.	Treatment of pediatric relapsed or refractory acute lymphoblastic leukemia after at least two prior chemotherapy regimens	[73,74]
Fludarabine	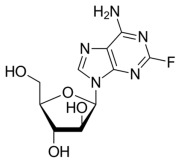	Fludarabine inhibits DNA polymerase, ribonucleotide reductase, and DNA primase. It is phosphorylated intracellularly to its active form, which incorporates into DNA, leading to chainTermination and apoptosis.	Chronic lymphocytic leukemia (CLL)Non-Hodgkinlymphoma	[75,76,77]
Nelarabine	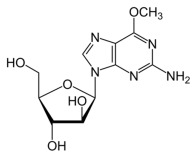	Nelarabine is a prodrug of 9-β-D-arabinofuranosylguanine which is phosphorylated intracellularly to its active form, ara-GTP. Ara-GTP incorporates into DNA, inhibiting DNA synthesis and inducing apoptosis in T-cells.	Treatment of relapsed or refractory T-cell acute lymphoblastic leukemia and T-cell lymphoblastic lymphoma	[78,79,80]

**Table 3:** Stereoelectronic features of purine and pyrimidine scaffolds.

Feature	Purine	Pyrimidine	Refs.
Structure	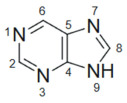	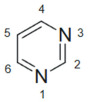	
Aromaticity and Planarity	Fused bicyclic aromatic system; planar	Monocyclic aromatic; planar	[90,91]
H-Bonding Sites	Multiple donors/acceptors (N1, N3, N7, N9)	Donors/acceptors at N1, N3 and C2/C4 substituents	
Electron Density Hotspots	C2 and C6 are reactive sites for substitution	C5 and C4 are typical modification points	[92]
Tautomerism	Yes—imidazole and pyrimidine ring tautomers	Yes—less pronounced than purines	[93,94]
Basicity/Acidity	Weakly basic (pKa ~ 2–4)	Neutral to weakly acidic	
Preferred Substitution Sites	C2, C6, N9	C2, C4, C5	
Molecular Geometry	Three-dimensional vector potential	Linear, compact structure	[95,96]
Interaction with DNA/Enzymes	Strong stacking with DNA bases; enzyme binding	Binds DNA/RNA via base mimicry; TS, DHFR targets	[97,98]

## Abbreviations

The following abbreviations are used in this manuscript:

5-FU	5-fluorouracil
AML	Acute Myeloid Leukemia
CDK	Cyclin-Dependent Kinase
c-KIT	Tyrosine-Protein kinase Kit
CML	Chronic Myeloid Leukemia
CSC	Cancer Stem Cell
DNA	Deoxyribonucleic Acid
EGFR	Epidermal Growth Factor Receptor
GIST	Gastrointestinal Stromal Tumor
HMEC	Human Mammary Epithelial Cells
MCF-7	Michigan Cancer Foundation-7
MDS	Myelodysplastic Syndromes
MTT	3-(4,5-dimethylthiazol-2-yl)-2,5-diphenyltetrazolium bromide
NSCLC	Non-Small Cell Lung Cancer
PBMC	Peripheral Blood Mononuclear Cells
PDGFR	Platelet-Derived Growth Factor Receptor
RNA	Ribonucleic Acid
ROS	Reactive Oxygen Species
THLE	Transeformed Human Liver Epithelial cells
TKI	Tyrosine Kinase Inhibitor
BCR-ABL	Breakpoint Cluster Region—Abelson Murine Leukemia Viral Oncogene Homolog 1
TrxR	Thioredoxin Reductase
TS	Thymidylate Synthase
